# Comparing Imaging Depth of Intravital Lung Imaging Using Perfluorocarbon‐Based Liquid Ventilation With Tissue Clearing for Deep‐Tissue Imaging

**DOI:** 10.1002/jbio.202500145

**Published:** 2025-09-03

**Authors:** Pascal Detampel, Wolf Heusermann, Katarzyna M. Wojcik, Bryan G. Yipp, Matthias Amrein

**Affiliations:** ^1^ Department of Cell Biology and Anatomy University of Calgary Calgary Alberta Canada; ^2^ Pharmaceutical Technology University of Basel Basel Switzerland; ^3^ Imaging Core Facility University of Basel Basel Switzerland; ^4^ The Snyder Institute for Chronic Diseases University of Calgary Calgary Alberta Canada; ^5^ Department of Critical Care Medicine University of Calgary Calgary Alberta Canada

**Keywords:** in vivo, intravital lung imaging, mice, multi photon microscopy, nanoparticle delivery, perfluorocarbon, refractive index, tissue optical clearing

## Abstract

Intravital lung imaging has been employed to study physiological and pathophysiological processes related to nanoparticle deposition in the alveolar lung, particularly in the context of air pollution and drug delivery. However, optical imaging depth is limited, often attributed to the refractive index (RI) mismatch at the alveolar air‐tissue interface. To investigate this, we evaluated two complementary strategies. First, we demonstrated that eliminating the RI mismatch via partial liquid ventilation with oxygenated perfluorocarbon (PFC) did not enhance the imaging depth. A second approach, utilizing ex vivo optical tissue clearing (with RI matching), was only successful in improving imaging penetration depth if it included removal of scattering lipids such as pulmonary surfactant. Nevertheless, partial liquid ventilation with PFC in vivo enabled the homogeneous delivery of nanoparticles to the alveoli, allowing real‐time observation of their interactions with lung epithelium. This finding opens new avenues for studying inhaled particulates and optimizing inhalation‐based drug delivery.

## Graphical abstract

Partial liquid ventilation with oxygenized perfluorocarbon (PFC) is used to assess the limitations of imaging depth, caused by the refractive index mismatch between air and surfactants in the mouse lung. PFC does not help to overcome this limitation. Only removing scattering lipids and decolorization ex vivo, with tissue optical clearing, enhance the penetration depth of optical imaging in the lung. Nevertheless, partial liquid ventilation with PFC allows for homogenously delivering and following nanoparticles to the alveoli.

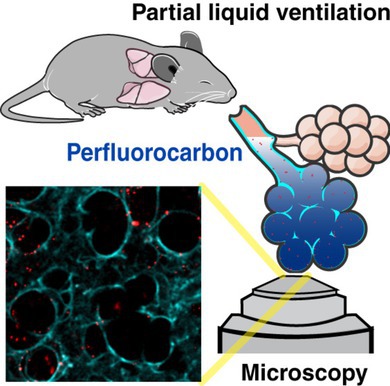

## 1 Introduction

The mammalian lung is responsible for the gas exchange of the blood, while it is exposed to inhaled microbes and (nano‐)particles from the environment. Therefore, it plays an important role in the immunological defense against inhaled pathogens [1, 2, 3, 4], explaining why immunological processes of the pulmonary system are currently intensely studied. High‐resolution intravital imaging of the pulmonary system has been notoriously difficult due to the movement of the breathing lung itself, coupled with contractions from the adjacent heart. In recent years, a variety of techniques have been developed which permit the imaging and recognition of various physiological and pathophysiological processes [5, 6, 7]. For example, neutrophils have been shown to rapidly respond and neutralize bacterial sepsis in the lung capillaries [4], and the role of macrophages in the lung on circulating tumor cells forming lung metastasis has been investigated [5, 8]. In addition, the potential of various pulmonary drug formulations as drug‐delivery systems to treat various lung diseases, such as inhaled nanomedicines, has been demonstrated [9]. However, the clearance of inhaled particles is still poorly understood [10], and a better understanding of their fate is a prerequisite for advances in targeted drug delivery to the lung.

Due to the optical properties of the lung, the imaging depth is limited to about 100 μm, even for multiphoton systems [6, 11]. This effect is attributed to the liquid‐air interface in the alveoli, which results in a strong scattering of the light due to a refractive index (RI) mismatch [7, 12]. However, many chronic lung diseases, such as asthma or primary lung cancer, require observation of pathophysiological dynamics in larger airways located deep in the lung tissue. Therefore, limited imaging depth has been considered a major limitation of current fluorescent‐based lung intravital imaging techniques [6, 7]. One way to circumvent the liquid‐air interface is by employing liquid ventilation and filling the lung with perfluorocarbon (PFC) [13]. This approach was successfully used with optical coherence tomography (OCT) and resulted in a substantial increase in imaging depth [14, 15]. If a similar improvement in imaging depth in the lung with fluorescence markers is achievable by replacing the air in the alveoli with PFC is unknown.

Another option to visualize deeper layers of an organ is by applying tissue optical clearing after removal from the body, albeit at the expense of losing the dynamic aspects present in a living organism. For example, by using an organic solvent to increase the RI and combining it with multiphoton microscopy, penetration depths of several mm were achieved in various mouse organs, including the lung [16]. Subsequently, various methods have been developed, all including RI matching, with different successes [17, 18]. While the physiological environment and dynamics of a living organism are lost, this ex vivo technique allows us to study and understand the optical challenges of the lung, especially in addressing the question of whether correcting an RI mismatch from the liquid‐air interface in the alveoli is sufficient to gain a greater imaging depth, which helps us better understand the limitations encountered when imaging in vivo.

The original goal of this study was to improve the image quality and the penetration depth of intravital lung imaging by filling the pulmonary system with PFC, to remove the liquid‐air interface in the alveoli, while maintaining oxygenation in vivo. Thus, we developed a protocol using partial liquid ventilation on an inverted microscope that permitted gravity‐assisted filling of the lung with perfluorotributylamine. Fluorescent nanoparticles suspended in PFC served to verify a successful filling of the alveoli with the fluorocarbon next to a proof of concept that this approach is also suitable to follow nanomedicines to the distal lung for drug delivery. Since removing the liquid‐air interface in the lung and increasing the RI did not improve the imaging depth significantly in vivo, we compared this with deep‐tissue imaging of the lung ex vivo with organ‐clearing methods and light‐sheet microscopy. We specifically compared two clearing protocols with similar RI, one with and one without delipidation and decolorization, to assess their impact on removing pulmonary lipids, such as surfactants, on imaging depth. This approach allowed us to observe that the liquid‐air interface in the alveoli and the RI mismatch are not the only factors limiting the imaging depth of intravital lung microscopy.

## 2 Materials and Methods

### 2.1 Intravital Lung Imaging and Application of Fluorescent Silica Particles

**FIGURE 1:**
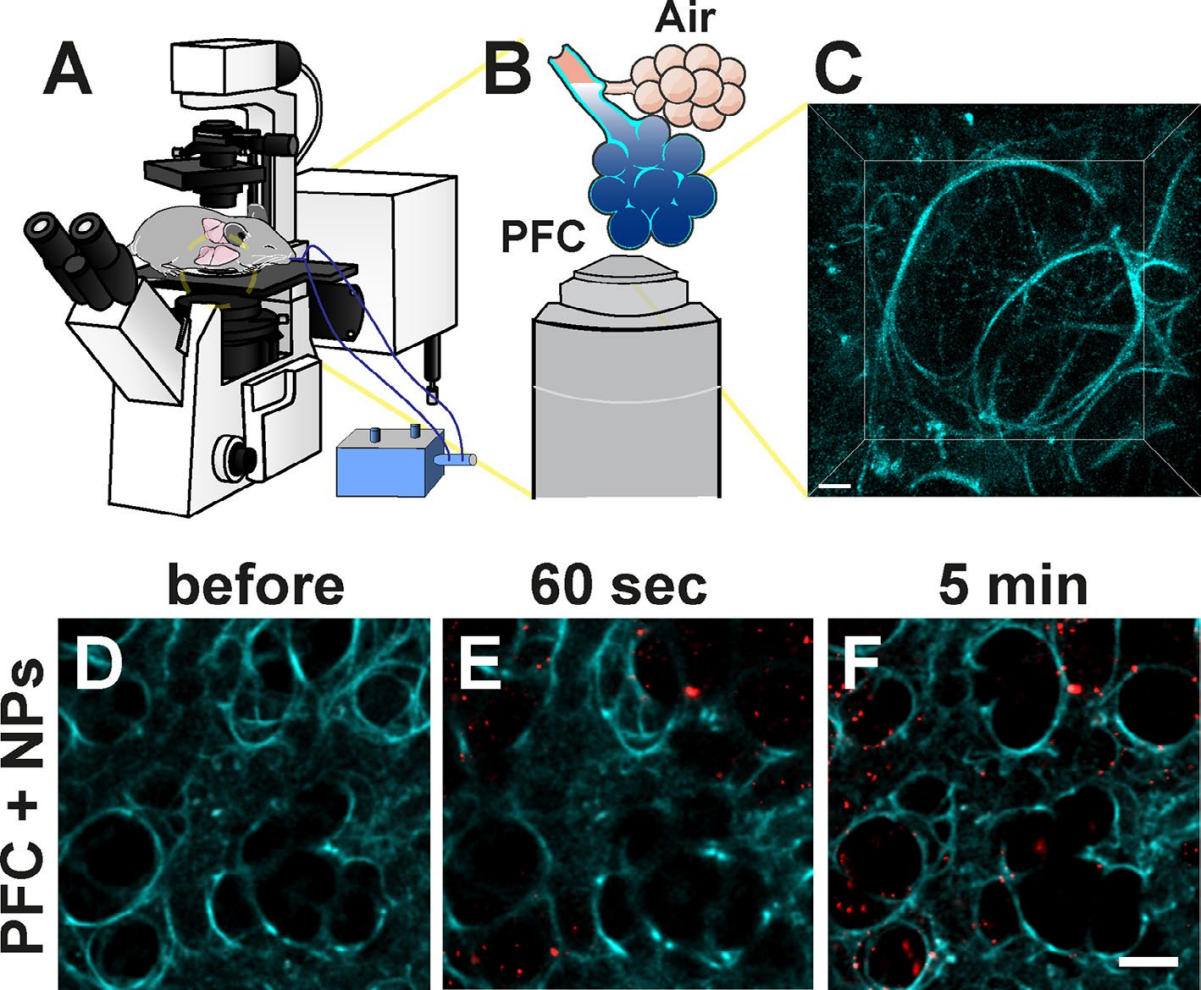
Schematic representation of partial liquid ventilation to remove the liquid‐air interface in intravital lung imaging (A–C), and visualization of perfluorocarbon (PFC)‐filled alveoli with fluorescent nanoparticles (NPs) (D–F). Setup rendering of a partial liquid‐ventilated mouse on an inverted microscope. Microscope adapter is not visualized (A). Illustration of alveoli filled with PFC for improved imaging (B). Representative image of a 3D view showing the autofluorescence (cyan) of alveoli filled with PFC (C). Representative image of intravital lung imaging of lung tissue before (D) and after instillation of fluorescent silica nanoparticles (red) suspended in PFC (E and F). To ensure successful delivery of PFC to the alveoli, fluorescent nanoparticles were visualized in vivo after administration (at 60 s, E) and were tracked over time (at 5 min, F). Scale bar (C): 10 μm, (D–F): 50 μm.

**FIGURE 2:**
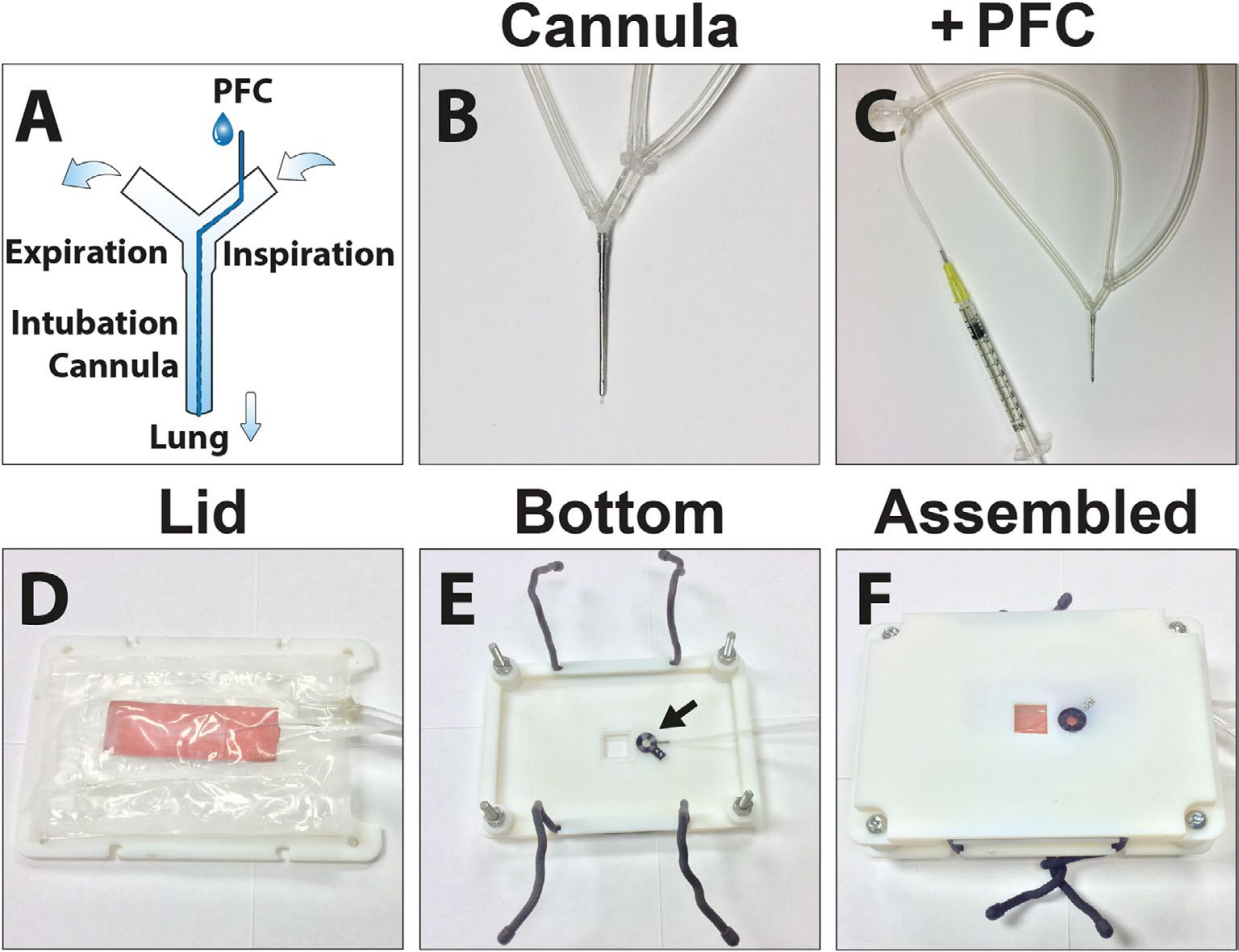
Design of a ventilation cannula with tubing incorporated for perfluorocarbon (PFC) instillation (A–C), and design of a microscopy stage adapter for inverted intravital lung imaging (D–F). Schematic representation of the ventilation cannula with the connections for the air flow. Inserted is a fine inner tubing permitting the instillation of PFC during ventilation (A). Photograph of ventilation cannula (B) and a zoomed‐out photograph displaying the connected syringe with PFC (C). Stage adapter lid combined with an inflatable air cushion and a heating pad (orange) (D). The bottom of the stage adapter with attached suction window (arrow) (E). The assembled and secured stage adapter with an inflated air cushion, used to stabilize the mouse and to enable mouse inversion for imaging on an inverted microscope (F).

Female C57BL/6 mice (protocol number AC14‐0105) were purchased from Jackson Laboratory (Bar Harbor, ME) and lysozyme M (LysM)‐eGFP mice (protocol number AC 14‐0038) were provided by T. Graf at the age between 8 and 16 weeks. All experiments were approved by the University of Calgary Animal Care Committee. Our approach for intravital lung imaging was aimed at eliminating the liquid‐air interface by partial liquid ventilation in a mouse (Figure 1) following a method reported by Looney et al. [19] and described previously [10]. In brief, animals were anesthetized by an i.p. injection of Ketamine (150 mg/kg) and Xylazine (10 mg/kg). The left jugular vein was cannulated for central venous access and used to administer anesthetic. A ventilation cannula for positive pressure ventilation (thin‐walled metal tube, 30 mm length, outer diameter 1.2 mm, inner diameter 0.8 mm) was inserted into the trachea and tied to the trachea. A PE‐10 tube was installed inside the ventilation cannula, through a side‐port, to allow the administration of PFC while simultaneously ventilating the mouse on the stage (Figure 2A–C). Mice were ventilated using a ventilator (MiniVent, Hugo Sachs Elektronik‐Harvard Apparatus, March‐Hugstetten, Germany) with a respiratory rate of 120 breaths per minute, a stroke volume of 200 μL, and a positive‐end expiratory pressure (PEEP) of 3 cm H_2_O.

A custom‐designed microscope insert for the inverted lung imaging approach was constructed (Figure S1) using the CAD SolidWorks software Version 2013 SP4 (Dessault Systèmes, Vélizy‐Villacoublay, France) and produced in VeroWhite RGD525 utilizing a Connex 500 Objet 3D printer with the software Objet Studio Ver. 9.0.10.19 (Stratasys, Eden Prairie, MN). The stage adapter was stabilized by an adjacent plate in a sandwich‐like assembly. A thoracic suction window was embedded into the stage, which allowed the inversion of the surgically prepared mouse on an inverted microscope (Figure 2D–F). This construction ensured that the cover glass touching the lung was always perpendicular to the axis of the objective while the mouse remained securely positioned. To attach the opposite plate to the microscopy stage, rubber cable ties were used to enable simple and fast fixation of the whole setup. The stage adapter was combined with an inflatable air cushion to immobilize the animal while rotating for inverted imaging. This cushion was built using a folded 0.1 mm Polyethylene sheet, which was sealed by ironing between parchment papers. A tube was connected using Plastic Bonder (J‐B Weld, Sulphur Springs, TX). Mice were placed in the right lateral decubitus position on an inflatable air cushion above the stage adapter lid (Figure 2D). The thorax was opened to have direct access to the lung. A thoracic suction window, with a 12 mm coverslip (#1.5), was screwed to the bottom part of the microscopy stage adapter (Figure 2E) and lowered upside down so that the coverslip was positioned above the lung. After assembling the microscopy insert (Figure 2F) and the lid of the stage adapter together with reusable cable ties (Nite Ize, Boulder, CO), the position of the cushion was re‐adjusted, and the cushion was carefully inflated until the lung contacted the suction window using a vacuum pump (DOA‐P104 AA, Gast Manufacturing Inc., Benton Harbor, MI) at ~130 mmHg. This setup enabled the mouse to be turned upside down, with the suction window attached to the lung, onto an inverted microscope while ventilating. In addition, a heating pad was included on the back of the adjacent plate of the microscopy stage adapter (Figure 2D) to keep the animal at 37°C while under deep anesthesia. The assembled mouse stage was positioned onto an inverted Nikon A1R MP+ microscope (Nikon, Melville, NY) with a resonant scanner and a Chameleon multiphoton laser (Coherent, Santa Clara, CA) equipped with a 40× water‐immersion long‐working distance objective (NA 1.15). Genteal artificial tears (Alcon, Mississauga, Canada) were used for water immersion between the coverslip and objective to reduce the surface tension between the objective and coverslip.

After focusing on a region of choice, the lung was ventilated with pure oxygen. Thereafter, ventilation was stopped for 60–90 s, which allowed the gaseous phase in the alveoli to disappear, before administering 120–150 μL of oxygenated perfluorotributylamine (FL‐43, from 3M, London, Canada) alone or combined with suspended 50 nm red fluorescent silica particles (PSi‐R0.05, excitation/emission: 569/585 nm, from Kisker Biotech, Steinfurt, Germany). The administration was instilled through the PE‐10 tubing inside the ventilation cannula. Silica particles were suspended at 500 μg/mL in PFC by dispersing 3× for 30 s with a Sonic Ruptor 250 tip sonicator (Omni International, Kennesaw, GA). Intravital images were taken with Nikon NIS‐Elements AR 4.20 software, and contrast adjustments were performed with ImageJ 1.50e software [20]. We acquired at least 3 data sets for each experiment and displayed a representative image.

### 2.2 Tissue Optical Clearing and Staining

Tissue optical clearing was performed based on the SeeDB [21] and CUBIC [22] protocols and after immunostaining. Isolated mouse lungs were fixed in ice cold 4% PFA (EMS, Hatfield, PA) in phosphate buffered saline (PBS) (Sigma‐Aldrich, Buchs, Switzerland) and Pen‐Fix (ThermoFisher Scientific, Waltham, MA) overnight at 4°C. After multiple washings in PBS, samples were blocked at 37°C in blocking solution containing 1% fetal bovine serum (FBS) and 0.1% Triton X‐100 (Sigma‐Aldrich) in PBS. Antibody‐stained samples were labeled 1:50 with anti‐alpha smooth muscle actin antibody (ab5694 from Abcam, Cambridge, UK) in blocking solution at 37°C with gentle rocking (90 RPM) for 11 days. Thereafter, immunolabeled lungs were washed with PBS, blocked overnight with blocking solution, and stained 1:500 with chicken anti‐rabbit Alexa 647 (A‐21443 from ThermoFisher Scientific) in blocking solution at 37°C with gentle rocking for 7 days. After multiple washings in PBS with 0.1% Triton X‐100, all lung sections were incubated according to the individual optical tissue clearing protocol. In brief, for SeeDB, samples were immersed gradually in fructose (Sigma‐Aldrich) solutions of 20%, 40%, 60%, 80%, and 100% (w/v) with the addition of 0.5% (v/v) α‐thioglycerol (Sigma‐Aldrich). Where applicable, nuclear staining was performed by adding 2 μg/mL propidium iodide (PI) to the fructose solution. Finally, RI matching was achieved by incubation in a saturated fructose solution of 80.2% (w/w) with the addition of 0.5% (v/v) α‐thioglycerol for 6 days. CUBIC‐cleared samples were incubated in CUBIC 1 solution 25% (w/v) urea (Nacalai Tesque, Japan), 25% (w/v) N,N,N′,N′‐tetrakis(2‐hydroxypropyl)ethylenediamine (Tokyo Chemical Industry CO, Japan), and 15% (w/v) Triton X‐100 (Nacalai Tesque, Japan) for 7 days, while the solution was changed after the first and third day. After multiple washings with PBS, RI matching was performed in CUBIC 2 solution (containing 50% (w/v) sucrose (Nacalai Tesque, Japan), 25% (w/v) urea, 10% (w/v) 2,2′,2″‐nitrilotriethanol (Wako Pure Chemical Industries, Japan), and 0.1% Triton X‐100) for a minimum of 24 h before imaging. When indicated, 2 μg/mL PI was added to the RI‐matching solution for nuclear labeling. The RI was measured on a Reichert AR7 Series refractometer (Reichert, Depew, NY) at 25.0°C.

### 2.3 Light‐Sheet Microscopy and Image Processing

Lung sections were immersed in the corresponding clearing solution and imaged with dual‐side illumination on a Zeiss Light‐sheet Z.1 microscope (Carl Zeiss, Jena, Germany), equipped with a 5× illumination (NA 0.1 foc.) and 5× EC Plan‐Neofluar detection (NA 0.16) objective. Fluorophores were excited using 488 and 638 nm lasers, coupled with a BP 505–545 nm and LP 640 nm emission filter, respectively. Image acquisition and light‐sheet processing were accomplished with ZEN 2014 SP1 (Black) and ZEN 3.7 (Blue) applications (Zeiss). Further visualization and analyses were performed with Imaris 9.9 (Oxford Instruments, Abingdon, UK) and ArivisVision4D version 3.1.1 (Zeiss) software.

## 3 Results

### 3.1 Establishing Intravital Lung Imaging With Liquid Ventilation and Ensuring the Alveoli Filling With Nanoparticles by Utilizing PFC

To ensure that the region of the lung that was fixed for imaging was totally filled with PFC, we utilized an inverted imaging setup to take advantage of gravity to fill the alveoli. In contrast to an upright microscope, the application of liquid ventilation always has the risk of imaging trapped air in alveoli when not all residual air is replaced with PFC. After successfully mounting the mouse on an inverted microscope and focusing on a region of interest on the lung surface, the pulmonary system was partially filled with PFC. To ensure complete infiltration with the fluorocarbon down to the lower alveoli, we ventilated the mouse with 100% oxygen for several minutes first and stopped ventilation for 60–90 s before instillation of PFC afterwards. This procedure led to a collapse of the alveoli as oxygen dissolved rapidly in the blood, making the alveoli accessible to the liquid. Ventilating with normal air resulted in insufficient delivery of PFC to the alveoli (data not shown). This was likely related to the high proportion of approximately 78% of nitrogen in air which, unlike oxygen, is poorly soluble in blood and prevented the PFC from reaching the distal part of the alveoli.

The filling of alveoli was evaluated based on our previous results where we investigated the clearance of nanoparticles in the deep lung [10]. Red‐fluorescent silica nanoparticles suspended in PFC were used to visualize a successful appearance of the fluorocarbon in the distal part of the lung (Figure 1D–F). Due to the strong autofluorescent properties of the lung tissue, especially in the shorter spectrum of the visible light, the structures of the alveoli were easily highlighted without any additional fluorescent marker (Figure 1C). The influx of PFC into the alveoli, visualized by suspended fluorescent silica nanoparticles (or their agglomerates), could be followed in real time (Video S1).

The number and distribution of nanoparticles increased during the first few minutes, which indicated that the process of filling the alveoli with PFC required some time to equilibrate (Figure 1E,F). In addition, we observed that the employed silica nanoparticles adhered to the alveolar wall while the lumen was filled with PFC. Hence, an even distribution of red‐fluorescent nanoparticles indicated a homogeneously filled distal part of the lung with PFC. Thereafter, a reduction of individual fluorescent agglomerates was detected over time (Video S1). This observation was linked to a partial clearance of the silica nanoparticles, while the lumen of the alveoli remained filled with PFC [10] due to gravity and the low surface tension of perfluorotributylamine.

While the focus of this work was on the imaging depth of partial liquid ventilation with PFC in mice, these results provided a proof of concept on how delivery of nanoparticles can be analyzed in the distal lung in real time. Next to the cellular distribution, the early fate of fluorescent‐labeled particles can be monitored in the alveoli in a living mouse. In our case, the visualization of suspended and fluorescent nanoparticles validated a homogeneous delivery of PFC in the distal part of the lung, which reduced the RI mismatch compared to air‐filled alveoli.

### 3.2 Imaging Depth Was Limited Even With In Vivo PFC‐Filled Alveoli

**FIGURE 3:**
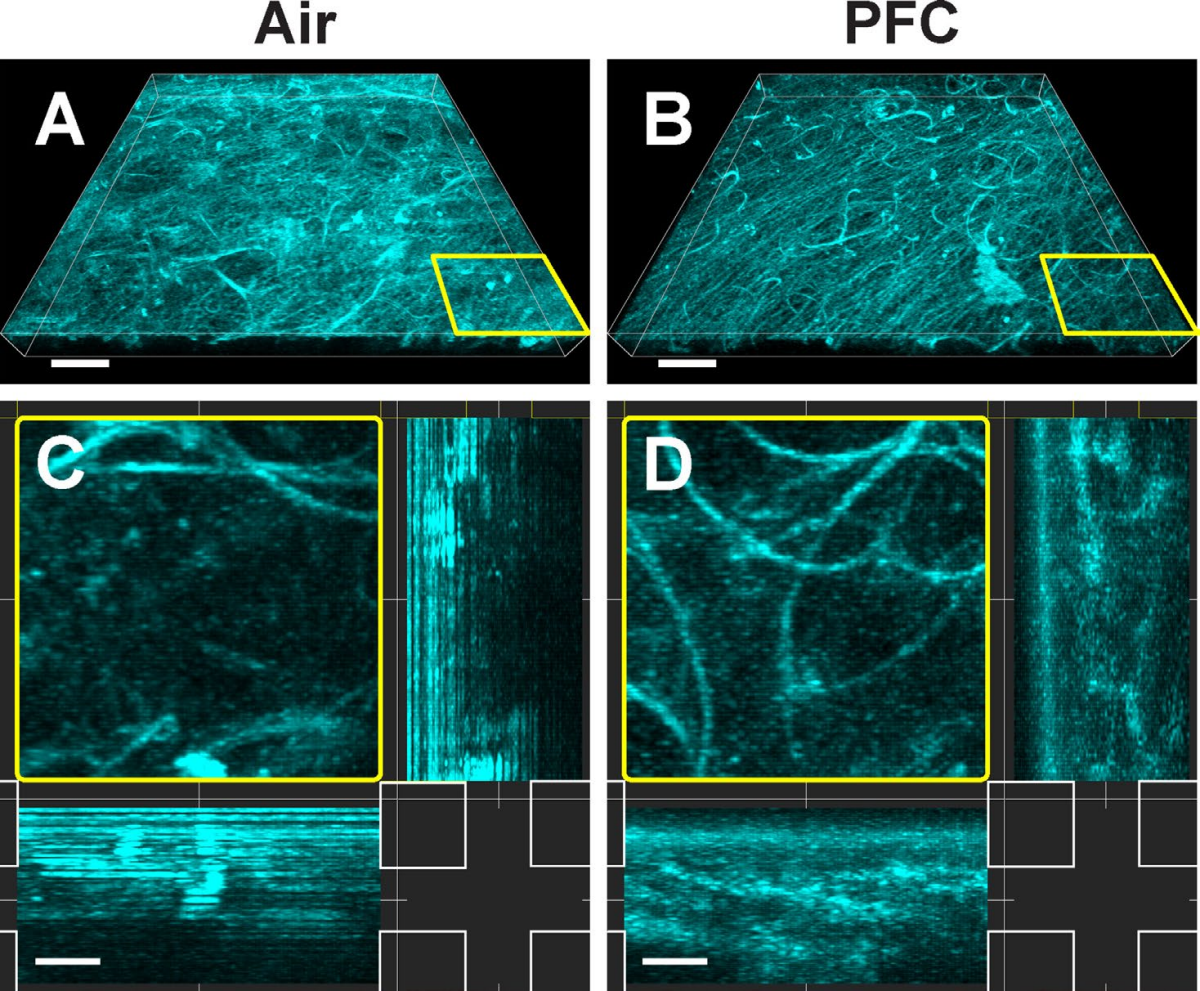
Comparison of intravital lung imaging of alveoli filled with air (A, C) to those filled with perfluorocarbon (PFC) (B, D). Representative confocal image of autofluorescence (cyan) of a z‐stack (27 μm) of lung tissue filled with air (A) in comparison to a similar section after partial liquid ventilation with PFC (B). Orthogonal view of an enlarged part (yellow frames) of the z‐stack of lung tissue filled with air (C) and filled with PFC (D). Note the improved visibility of deeper lung structures with alveoli filled with PFC (B, D). Scale bar A and B: 50 μm; C and D: 10 μm.

After ensuring that the alveoli were filled with PFC, we investigated how the removal of the liquid‐air interface in the lung and the increase in RI from 1.00 (air) to 1.291 (perfluorotributylamine [23]) affected the imaging quality. First, a z‐stack of the outer region (27 μm) of the autofluorescent lung tissue filled with air versus perfluorotributylamine was compared using confocal microscopy (Figure 3). In general, a reduced scattering of the outer lung surface was noticeable (Figure 3A,B). While superficial structures of the air‐filled lung showed a strong autofluorescent signal, the PFC‐filled area displayed a more homogeneous illumination with confocal microscopy. Especially in the orthogonal view, the increased and more uniform imaging depth was visible in the alveoli filled with perfluorotributylamine (Figure 3C,D). Hence, for the first two dozen μm of penetration depth, removing the liquid‐air interface and the increase in RI were beneficial for image quality using laser‐scanning microscopy. While nanoparticles were easily delivered and imaged in the distal part of the lung with PFC (Figure S2), we were not able to detect them after instillation, which is another common method of delivering nanoparticles to the lung [24].

**FIGURE 4:**
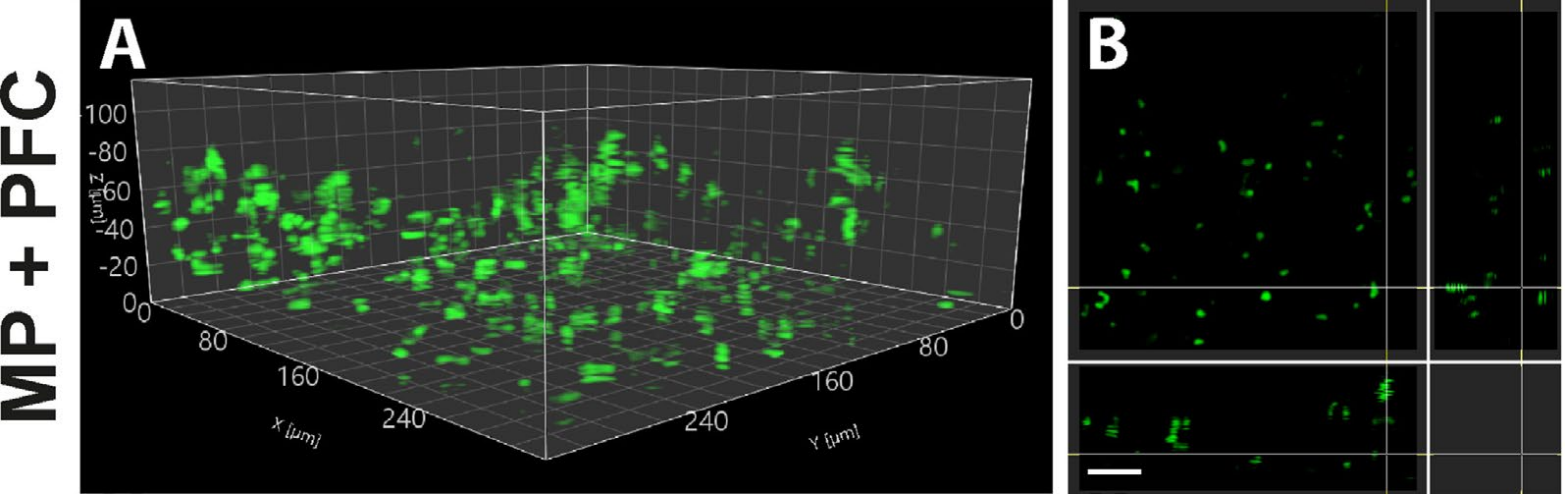
Multiphoton (MP) microscopy of neutrophils in the lung with perfluorocarbon (PFC)‐filled alveoli. Representative image of a 3D view of eGFP (green) expressing neutrophils (LysM‐eGFP) in a mouse lung using multiphoton excitation at 860 nm (A) and the corresponding orthogonal view of the *z*‐stack (B). Note that the imaging depth is limited around 115 μm with PFC‐filled alveoli in the lung. Furthermore, only a single fluorescence channel is displayed, and no anatomical reference or alveoli are visible. Scale bar B: 50 μm.

Next, we examined the fluorescence signal and the penetration depth utilizing two‐photon‐excited fluorescence to visualize eGFP expressing neutrophils in a PFC‐filled lung from lysozyme M (LysM)‐eGFP mice (Figure 4). Neutrophils were clearly visible and displayed a uniform signal throughout the field of view (Figure 4A). However, the multiphoton z‐stack was limited to about 115 μm imaging depth even though the liquid‐air interface was removed (Figure 4B). Furthermore, the contrast of the signal of the two‐photon images had to be normalized depending on the z‐plane to enhance the signal to visualize the maximum imaging depth. In summary, while filling the lung with perfluorotributylamine resulted in an even illumination of autofluorescent structures and fluorescent‐emitting molecules, the penetration depth was still limited below the surface of the lung tissue. Therefore, even by utilizing two‐photon fluorescence microscopy combined with PFC‐filled alveoli, the signal from eGFP expressing neutrophils was still restricted in the range of 100 μm imaging depth.

### 3.3 Delipidation Was Required to Improve Deep Lung Imaging

Since removing the liquid‐air interface and increasing the RI in the lung with PFC did not result in a significant extension in imaging depth in vivo, we wanted to compare different ex vivo clearing methods of the lung—all using a similar increased RI—to better understand the limiting factors for deep‐tissue imaging of the pulmonary system. Next to an increased RI, we wanted to investigate the role of pulmonary surfactant, which is mostly composed of lipids [25] and endogenous chromophores on imaging penetration depth. Hence, we chose to compare a simple aqueous‐based immersion technique with a high RI clearing solution, such as SeeDB [21], versus a method that utilizes delipidation and decolorization in a hyperhydrating environment followed by immersion in a high RI solution, such as CUBIC [26].

An overview, on a macroscopic level, of the different cleared lung sections after fluorescent labeling was acquired (Figure S3). While the transparency of the lung tissue immersed in the SeeDB solution did not increase, a noticeable difference was seen after clearing with CUBIC‐1 and immersing in a CUBIC‐2 solution. As the CUBIC solutions resulted in a decolorization of the tissue, the samples stained with propidium iodide displayed a reddish color, probably from the dye itself.

**FIGURE 5:**
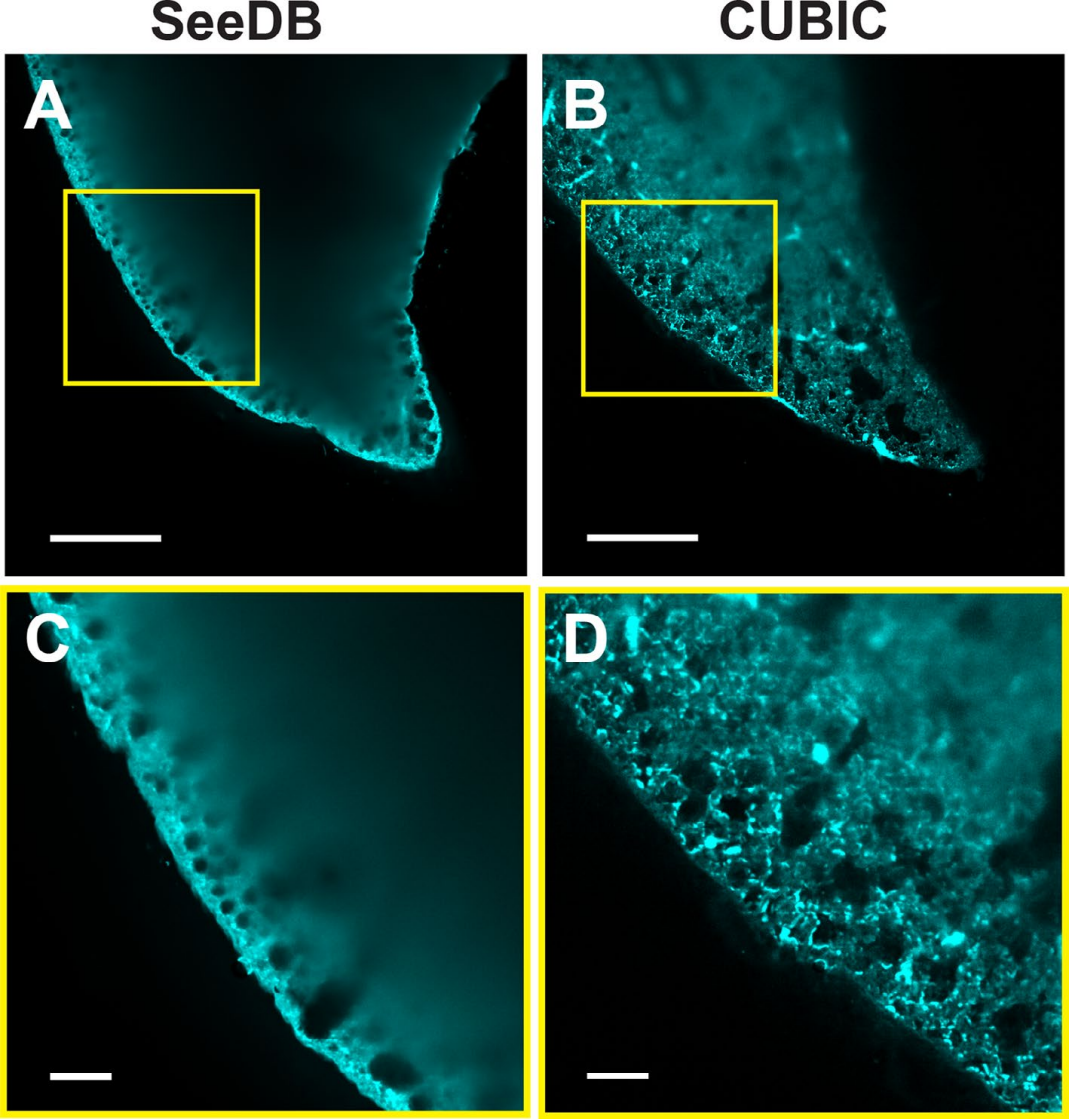
Ex vivo deep‐tissue imaging of fixed mouse lungs after organ clearing without and with delipidation and decolorization using light‐sheet microscopy. Autofluorescent lung tissue (cyan) is visualized after a refractive index‐matching lung clearance protocol (SeeDB—RI: 1.485) (A) and compared to a clearing method employing additional high concentrations of detergents and decolorants (CUBIC—RI: 1.484) (B). Enlarged views of the lung surfaces of SeeDB and CUBIC‐cleared lungs are shown in (C) and (D), respectively. Scale bar A and B: 500 μm, C and D: 100 μm.

Next, we assessed the imaging depth of the SeeDB and CUBIC‐cleared lung tissues, which shared a very similar RI, using a light‐sheet microscope (Figure 5). The RI of the fructose‐based SeeDB solution was 1.485, while the sucrose‐based CUBIC‐2 solution had an RI of 1.484. By imaging the autofluorescent lung tissue, a significant improvement in deep‐tissue imaging was notable in the delipidated and decolorized CUBIC sample, whereas only an unscattered signal was received from the surface of the SeeDB‐cleared lung (Figure 5A,B). When zoomed in, the differences in penetration depth were emphasized. As the fluorescent signal got blurry beyond 50 μm depth in the SeeDB sample, the autofluorescent structures were clearly visible several hundred μm into the CUBIC‐cleared tissue when the organ was optimally positioned within the light‐sheet illumination (Figure 5C,D).

**FIGURE 6:**
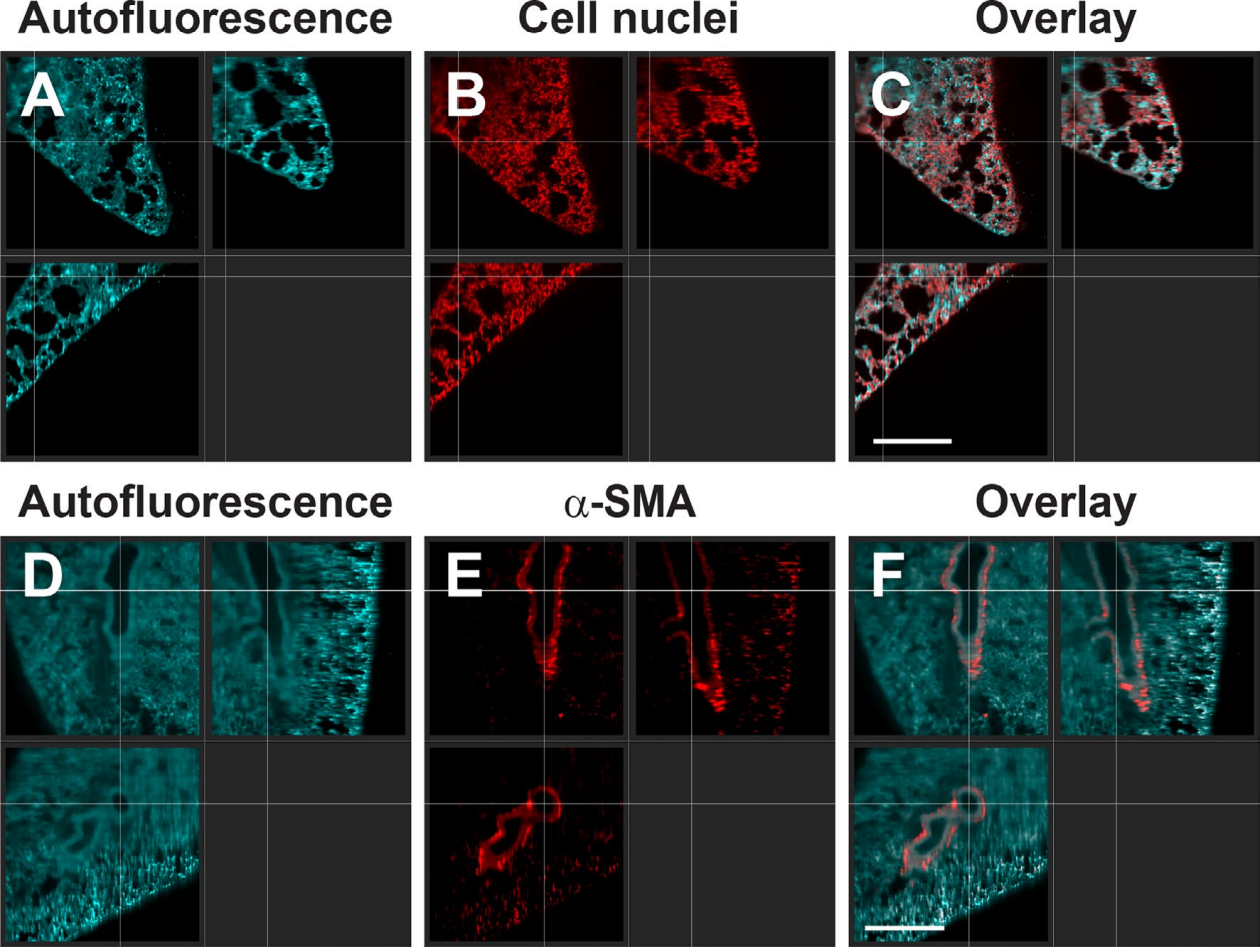
Light‐sheet microscopy of deep lung tissue after delipidation, decolorization (CUBIC), and staining with different fluorescent labels. Imaging of ubiquitous cellular components, such as nuclei, throughout the lung: Autofluorescence—Cyan (A), cell nuclei (propidium iodide)—Red (B), and the matching overlay (C). Staining of inner structures, such as alpha smooth muscle Actin (α‐SMA) from the lung: Autofluorescence—Cyan (D), anti‐alpha smooth muscle antibody (Alexa 647)—red (E), and the corresponding overlay (F). Scale bars: 500 μm.

Subsequently, we complemented these data with imaging two types of stains in deeper lung structures after delipidation and decolorization (CUBIC). The small nuclear dye propidium iodide was used to label nuclei in the tissue (Figure 6A–C). Next to the autofluorescence, a homogeneous pattern of the DNA‐intercalating dye was visible throughout the lung tissue. Afterwards, an antibody staining against alpha smooth muscle actin (Anti‐α SMA) in the trachea and arteries in the lung was imaged (Figure 6D–F). Here, smooth muscle structures were clearly visible in the central part of the lung alongside the autofluorescent tissue. A 3D‐reconstructed animation of stained smooth muscle cells of the pulmonary arteries and trachea is provided in Video S2. Hence, delipidation and decolorization in combination with RI matching were required for deep lung imaging.

## 4 Discussion

Intravital lung imaging has revealed a plethora of different capabilities to follow and understand various physiological and disease processes in the lung. Already in the 17th century, Marcello Malpighi used an early version of a microscope to study the lungs of living frogs, which led to the discovery of pulmonary capillaries and alveoli [27]. Only in the last few decades has it become possible to perform intravital lung imaging also in mammals. Especially investigations of the mouse lung have revealed deep insights into physiological and pathophysiological mechanisms [6]. For example, significant progress in understanding the function of various immune cells in the lung, such as neutrophils at steady state, as well as in inflammatory reactions and multiple stages of lung metastasis, including arrival, extravasation, and progression, has been made [4, 11, 28]. However, one substantial limitation of intravital lung imaging is the limited imaging depth to around 100 μm, which prevents the analysis of deeper chronic airway diseases like asthma. This restriction has been repeatedly linked to the RI mismatch of the liquid‐air interface of the alveoli and the lung tissue [6, 7, 11, 29]. Hence, reducing the RI mismatch should improve the quality of deep‐tissue imaging and expand the imaging depth in the lung.

One approach to remove the liquid‐air interface is to fill and ventilate the lungs with PFC which has an RI close to the lung tissue. Our rationale, when testing this approach, was to reduce the RI mismatch between the air in the alveoli (RI of 1.00) and the lung tissue (RI range between 1.30 and 1.41) [30]. Therefore, we administered perfluorotributylamine, with an RI of 1.291, into the lungs through a tracheal cannula. A similar approach has been successfully utilized with magnetic resonance imaging, enhancing the contrast in the lung [31] and with optical coherence tomography (OCT), where near‐infrared light is used by interferometry to obtain cross‐sectional structures of tissues [15]. By applying OCT combined with total liquid ventilation in mice, more than a threefold increase in imaging depth of up to 700 μm was achieved by Schnabel et al. [14, 15]. This approach uses an upright microscopy setup where the thorax window is located at the highest point of the lung and, thus, total liquid ventilation is required to completely fill the imaged subpleural lung structures with PFC. Next, to adapt this technique to optical fluorescence microscopy, our approach was to use an inverted microscope setup that permitted the use of partial liquid ventilation. Hence, only about 50%–60% of the functional residual lung capacity [32] was filled with PFC. The low surface tension (γ: 16 mN/m) of perfluorotributylamine [23], combined with the high weight density (ρ: 1.86 g/cm^3^), ensured that the lung region attached to the thoracic suction window at the bottom of the microscope stage was rapidly filled with the liquid by gravity. By using suspended fluorescent nanoparticles in PFC, which became visible shortly after instillation, we confirmed a successful and homogeneous filling of the lower alveoli. Another common option to deliver nanoparticles in an in vivo setting is the use of intratracheal instillation [24]. In our case, a successful deposition and imaging of the nanoparticles in the distal parts of the lung without PFC was not achievable, which might be linked to the fact that this method produces a highly inhomogeneous particle distribution in the lung [33, 34], next to the risk of creating an inflammatory response [35]. However, a detailed comparison of different nanoparticle‐delivery methods to the terminal alveoli, including immunological responses, would be important but is beyond the scope of the present study.

While some improvements in image quality were visible in the outer alveoli, the imaging depth was still restricted to the 100 μm range. Even the use of multiphoton microscopy with PFC‐filled lungs did not allow for the visualization of structures beyond this limit. These results were in contrast to penetration depths of optical imaging of other organs, such as the pancreas or brain, which are in the scale of 250 and 600 μm, respectively [36, 37]. Hence, removing the liquid‐air interface in the alveoli with PFC did not improve the imaging depth in the lung and is similar to previous air‐ventilated approaches [19]. Therefore, our results showed that the RI mismatch between air and tissue was not the only obstacle for deep lung imaging. Although the RI of perfluorotributylamine (RI 1.291) [23] was not a perfect match for the lung tissue (RI 1.30–1.41) [30], a substantial improvement to air (RI 1.00) was expected. Other PFCs that can be used for liquid ventilation, such as perfluor‐n‐octan or perflubron, have a lower or very similar RI of 1.282 and 1.296, respectively. Hence, no difference in imaging depth is expected compared to the perfluorotributylamine we used. Potentially, the multi‐layered pulmonary surfactant film [25, 38] that coats the alveoli to the air (or in our case to the PFC) contributes to the limiting penetration depth, specifically since the surfactant in the lungs consists primarily of lipids that have strong light‐scattering properties [39, 40].

To further investigate the role of the RI mismatch and how additional factors like scattering lipids affected the imaging depth of lung tissue, we conducted ex vivo studies with tissue optical‐clearing techniques combined with light‐sheet microscopy as a second method. First, the SeeDB protocol employed a simple fructose‐based immersion to increase the RI to about 1.49 inside the lung tissue and was successfully used for deep imaging of brain samples [21]. However, it was not sufficient to increase the penetration depth in the lung. Hence, we compared a second clearing protocol (CUBIC) with a very similar RI of 1.48–1.49 paired with delipidation and decolorization [26]. This time, the penetration depth was substantially increased. Thus, the organ transparency was not only linked to RI matching but also to delipidation and decolorization of the lung tissue [22, 41]. The role of large libraries of chemical compounds for delipidation and decolorization, and their potential use in clearing protocols for various organs, including the lung, has been investigated in depth by Tainaka et al. Our results were in line with their findings, which showed that RI matching is not sufficient for deep‐tissue imaging in the lung but also requires delipidation and decolorization [41]. Other clearing methods, such as BABB, 3Disco, and its derivatives, use an even higher RI in the range of 1.56 by immersion in organic solvents. Even though the RI is substantially above that from lung tissue (RI 1.30–1.41) [30], an even better transparency of the lung is achieved [18]. These organic solvent‐based clearing techniques permit the visualization by confocal microscopy of the morphology and subcellular structures of a complete mouse lung [42]. However, this approach is linked to significant organ shrinking, and the use of organic solvents adds limitations by signal‐quenching fluorescence proteins [17, 18]. Recently, another approach has been described where dyes with sharp absorption peaks can lead to augmented transparency in vivo by elevating the RI in the tissue through the Kramers‐Kronig relation [43]. After topical application or injection, followed by diffusion of these dyes in the tissue, blood vessels and the sarcomeres from muscle cells could be imaged several hundred micrometers through the skin. However, a significant RI mismatch in a tissue or organ, such as the lipid layer of the pulmonary surfactant on the alveoli, will lead to extensive light scattering with a reduced penetration depth, which limits its use for lung imaging.

Although the focus of this work was on improving the imaging depth with liquid ventilation in intravital lung imaging, we have also shown that the kinetics of suspended nanoparticles in alveoli can be followed in vivo. Detailed observations of nanoparticles translocating the lung epithelium and the clearance kinetics were described by us earlier, followed by reports on uptake and intracellular transport mechanisms across the alveolar epithelial barrier [10, 44]. Nevertheless, in general and especially in the lung, there is a need to better understand the barriers that prevent efficient organ drug delivery as well as cellular uptake of nanoparticles for diagnostic and therapeutic use. Intravital imaging can help to understand these processes [45, 46]. The choice of using PFC with intravital imaging has been shown to be safe and has several advantages. PFCs are considered to be chemically and biologically inert; specifically, perfluorotributylamine is a well‐tolerated PFC that has been used for in vivo imaging via various routes of application [47]. Further, utilizing partial liquid ventilation to transport nanoparticles to the lung solves problems reaching the distal alveoli, unlike intratracheal instillation [33, 48]. In addition, PFC has been shown to enhance external gene delivery and expression after viral and liposomal transfection in rodents and nonhuman primates without showing any signs of toxicity [49, 50]. Since we were able to follow the arrival and the dynamics of our nanoparticles in the alveoli for an hour, it serves as proof of concept that this technique can be employed to investigate and optimize nanomedicines for lung delivery.

However, our imaging technique is restricted to an observation time span of a few hours, due to the highly invasive nature of the procedure, which is generally a limiting factor of intravital lung imaging investigations [51]. Although perfluorotributylamine is considered as non‐toxic, possible influence on the natural behavior of pulmonary cells and neutrophils still remains to be investigated [52]. In general, implanting a vacuum‐stabilized imaging window, as we did, must be evaluated with caution. Especially since the vacuum can alter the natural physiology of the lung and cause localized tissue damage, notably at the border of the imaging window which needs to be excluded from image acquisition. Notably, the applied vacuum pressure, needed to stabilize the imaging window, proved to be critical for successful imaging. We found a value of ~130 mmHg below atmospheric pressure sufficient to arrest the lung firmly on the window, while blood flow in the vasculature was still permitted. Higher values of 260 mmHg below atmosphere prevented the opening of the alveoli, hindering PFC and nanoparticles from reaching the distal lung. However, Looney et al. reported an even lower vacuum [19], closer to physiological values in the pleura. We were not able to lower the pressure to that level, as the lung was not stable enough for imaging, and in fact would peel away from the coverslip on our window. Entenberg et al. used a different technique which avoided suction completely by utilizing cyanoacrylate adhesive to stabilize the window for long‐term mouse imaging [11].

Restricted imaging depth, as one of the major limitations of intravital lung imaging, remains a significant barrier to fully visualize lung physiology and dynamics. So far, most approaches image the lung from the distal pleural surfaces, which prevents the analysis of diseases located at larger airways and blood vessels. Only recently, a new method was reported to visualize pulmonary veins and surrounding lymphatic vessels deep in the tissue. This method uses a special 3D‐printed thoracic window and a modified surgical technique that allows imaging the lung from the mediastinal pleural surface and observing leukocyte migration and lymph flow through intact pulmonary lymphatics [28]. Although analyzing the lung from another angle reveals new insights, the restrictions regarding imaging depth remain unchanged.

Other techniques, like adaptive optics with machine learning [53] or use of deformable mirrors combined with multiphoton microscopy [54], are developed to increase imaging depth in the brain [55] and the bone marrow through the mouse skull [56]. It will be of interest to see if such approaches can also help to improve the penetration depth in a challenging optical environment such as the lung tissue.

## 5 Conclusion

In this publication, we described the procedure for partial liquid ventilation for intravital fluorescence microscopy of the mouse lung. Ventilating the animal with perfluorotributylamine, on an inverted microscope, led to the elimination of the liquid‐air interface within the alveoli, by gravity‐assisted displacement of air. However, removing the liquid‐air interface did not increase the penetration depth of optical imaging in the lung tissue in vivo, which indicated that other components within the lung, such as alveolar surfactants, were responsible for limiting imaging depths. To confirm this, we compared the SeeDB‐clearing protocol to the CUBIC‐clearing method in the lung tissue ex vivo. The SeeDB‐clearing protocol showed that RI matching by itself did not lead to an enhanced penetration depth of the alveoli. Only switching to the CUBIC‐clearing method, which combined RI matching with delipidation and decolorization, resulted in a significantly increased penetration depth. Although combining RI matching with delipidation and decolorization would be most effective, the use of the CUBIC‐clearing method is not feasible in vivo. While we did not show that eliminating the liquid‐air interface by using PFC improved signal penetration on the *z*‐axis, we showed that intravital imaging of nanoparticles in a mouse lung with liquid ventilation is possible. We anticipate that this approach can be applied to better understand the physiological and pathophysiological interactions of nanoparticles in the pulmonary system. Additionally, this approach may be used to facilitate insight into particle delivery, transport, and interaction with different cells in the lung.

## Conflicts of Interest

The authors declare no conflicts of interest.

## Supporting information


**Figure S1:** Technical drawing of the microscopy stage adapter used to stabilize the ventilated mouse lung on an inverted microscope.
**Figure S2:** Perfluorocarbon (PFC)‐filled alveoli with red‐fluorescent nanoparticles (NPs).
**Figure S3:** Transparency of ex vivo lung sections after employing different clearing and staining protocols.

**Video S1:** Intravital lung imaging showing the entrance of perfluorocarbon with suspended red‐fluorescent nanoparticles in the alveoli. A time lapse of confocal images displaying the arrival and evenly distribution of fluorescent nanoparticles (red) in the alveoli (autofluorescence—cyan) in the first 90 s (min: sec), Scale bar 50 μm.

**Video S2:** 3D animation of the trachea and the pulmonary arteries of a mouse lung lobe. Light‐sheet microscopy after staining alpha smooth muscle actin and subsequently applying tissue optical‐clearing protocol CUBIC. The 3D animation shows primarily the surface of smooth muscles of the distal part of the trachea from different angles, and a moving clipping plane reveals the inner structure.

## Data Availability

The data that support the findings of this study are available from the corresponding author upon reasonable request.
