# Supplementary material for: Comparing Imaging Depth of Intravital Lung Imaging Using Perfluorocarbon‐Based Liquid Ventilation With Tissue Clearing for Deep‐Tissue Imaging
Source: J Biophotonics. 2025 Sep 3;19(1):e202500145. doi: 10.1002/jbio.202500145 (PMC12809380; doi:10.1002/jbio.202500145)
Supplement: Supplementary file 1 — Figure S1: Technical drawing of the microscopy stage adapter used to stabilize the ventilated mouse lung on an inverted microscope. Figure S2: Perfluorocarbon (PFC)‐filled alveoli with red‐fluorescent nanoparticles (NPs). Figure S3: Transparency of ex vivo lung sections after employing different clearing and staining protocols. [file JBIO-19-e202500145-s002.docx]

## Supplementary Information

**Comparing Imaging Depth of Intravital Lung Imaging Using Perfluorocarbon-Based Liquid Ventilation With Tissue Clearing for Deep-Tissue Imaging**

*Pascal Detampel^1,2*^, Wolf Heusermann^3^, Katarzyna M. Wojcik^4^, Bryan G. Yipp^5^, Matthias Amrein^1*^*

^1^ Department of Cell Biology and Anatomy, University of Calgary, Calgary, Alberta, Canada

^2^ Pharmaceutical Technology, University of Basel, Basel, Switzerland

^3^ Imaging Core Facility, University of Basel, Basel, Switzerland

^4^ The Snyder Institute for Chronic Diseases, University of Calgary, Calgary, Alberta, Canada

^5^ Department of Critical Care Medicine, University of Calgary, Calgary, Alberta, Canada

Corresponding authors: Pascal Detampel ([pascal.detampel@unibas.ch](mailto:pascal.detampel@unibas.ch)) and Matthias Amrein ([mamrein@ucalgary.ca](mailto:mamrein@ucalgary.ca))


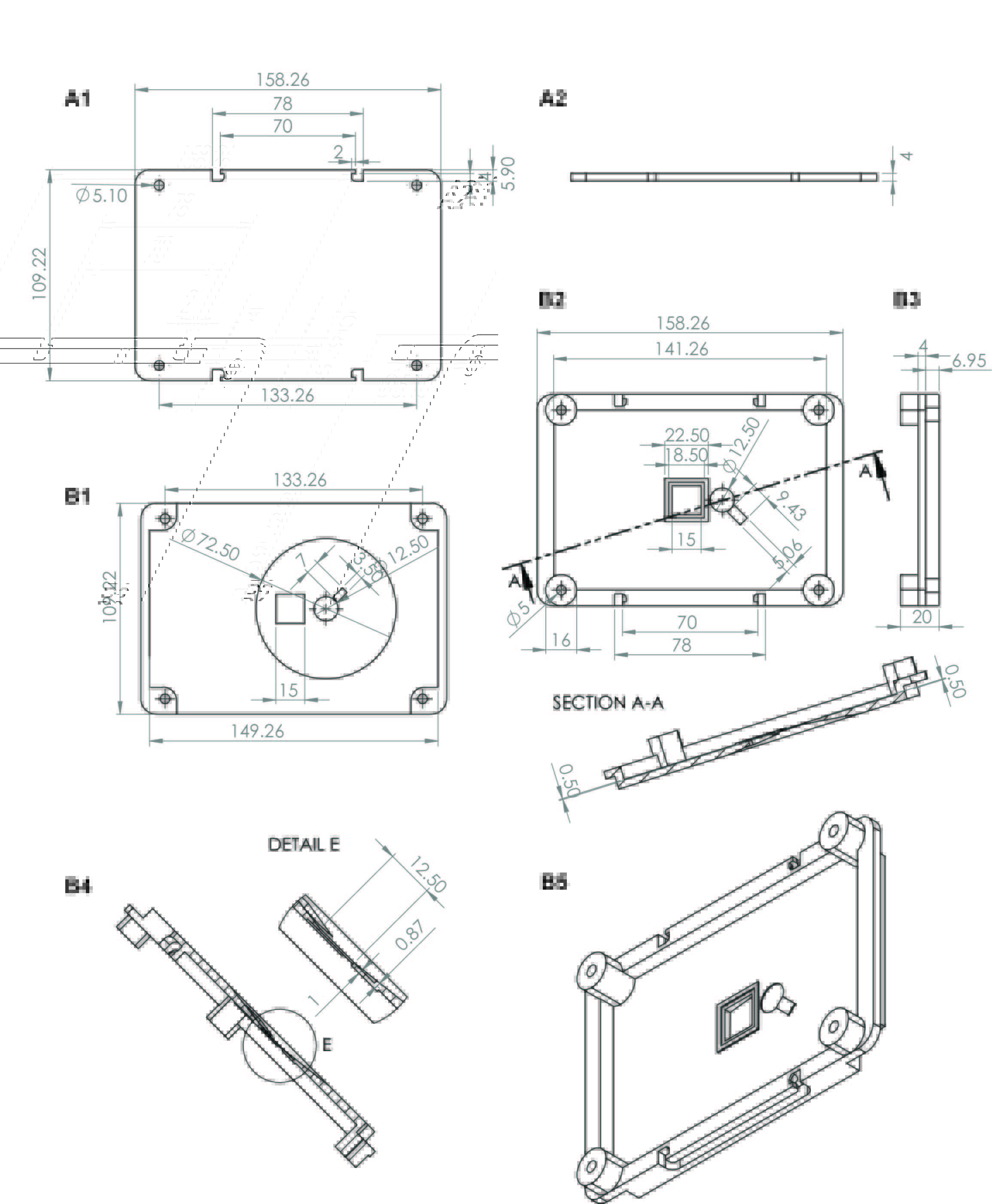


**Supplemental Figure S1:** Technical drawing of the microscopy stage adapter used to stabilize the ventilated mouse lung on an inverted microscope. Horizontal (A1) and frontal (A2) projection of the lid. Horizontal projection of the insert viewed from below (B1) and above (B2), combined with a side projection (B3). A sectional drawing of the area where the suction window is attached is presented (B4) together with a magnified overview from above (B5). Note, the suction window is screwed onto the 12.5 mm round opening, and the 15 mm square is covered with a cover glass to facilitate the positioning of the animal. All measurements are in mm.


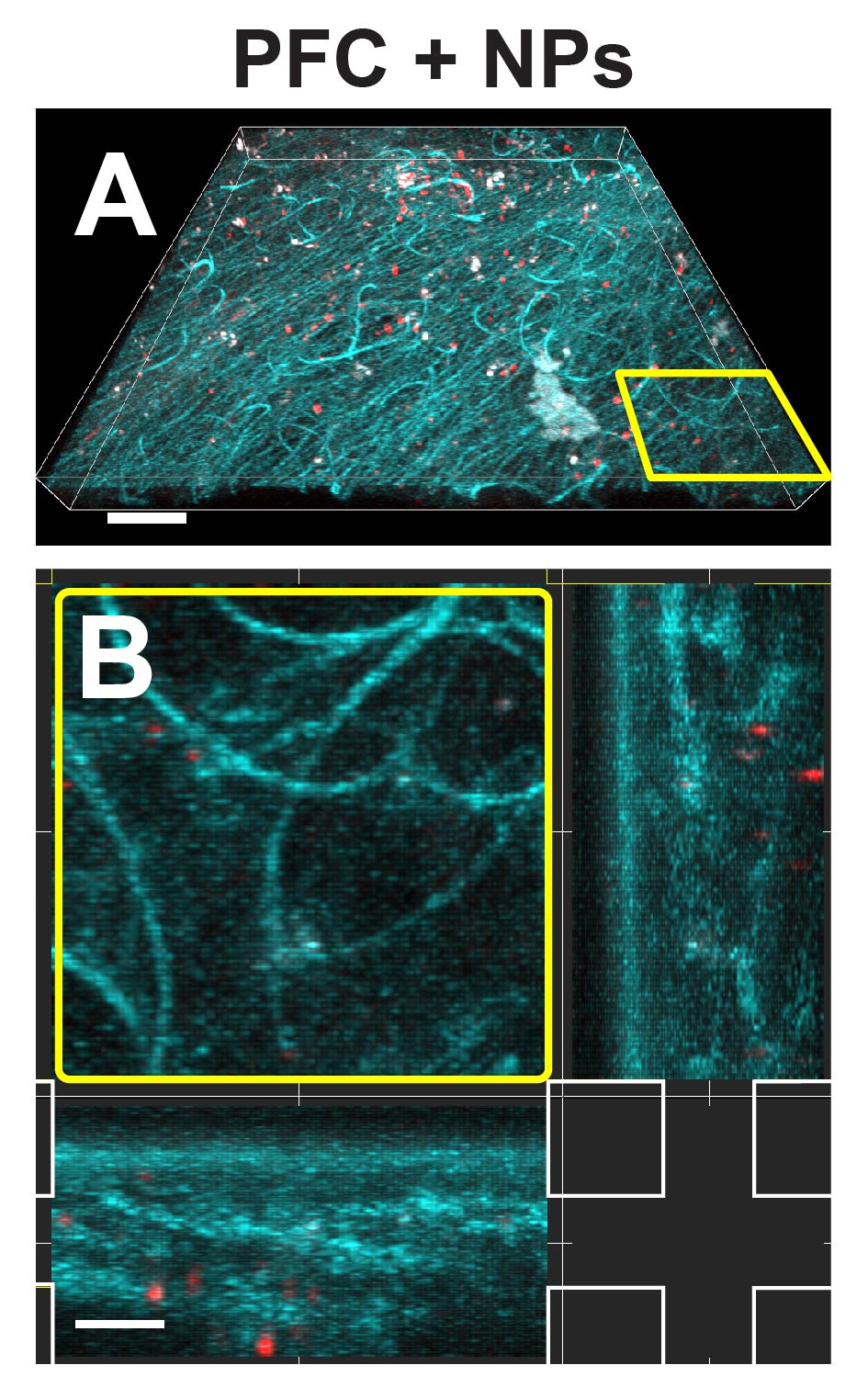


**Supplemental Figure S2, in addition to Figure 3:** Perfluorocarbon (PFC)-filled alveoli with red-fluorescent nanoparticles (NPs). Autofluorescence (cyan) of a z-stack (27 µm) of the distal part of the lung acquired with a confocal microscope (A). The corresponding orthogonal view of an enlarged part (yellow frame) of the z-stack of lung tissue (B). Note that a comparison with deposited NPs without PFC (air) was not achievable in the current setup, thus showing only NPs with PFC. Scale bar A: 50 µm; B: 10 µm.


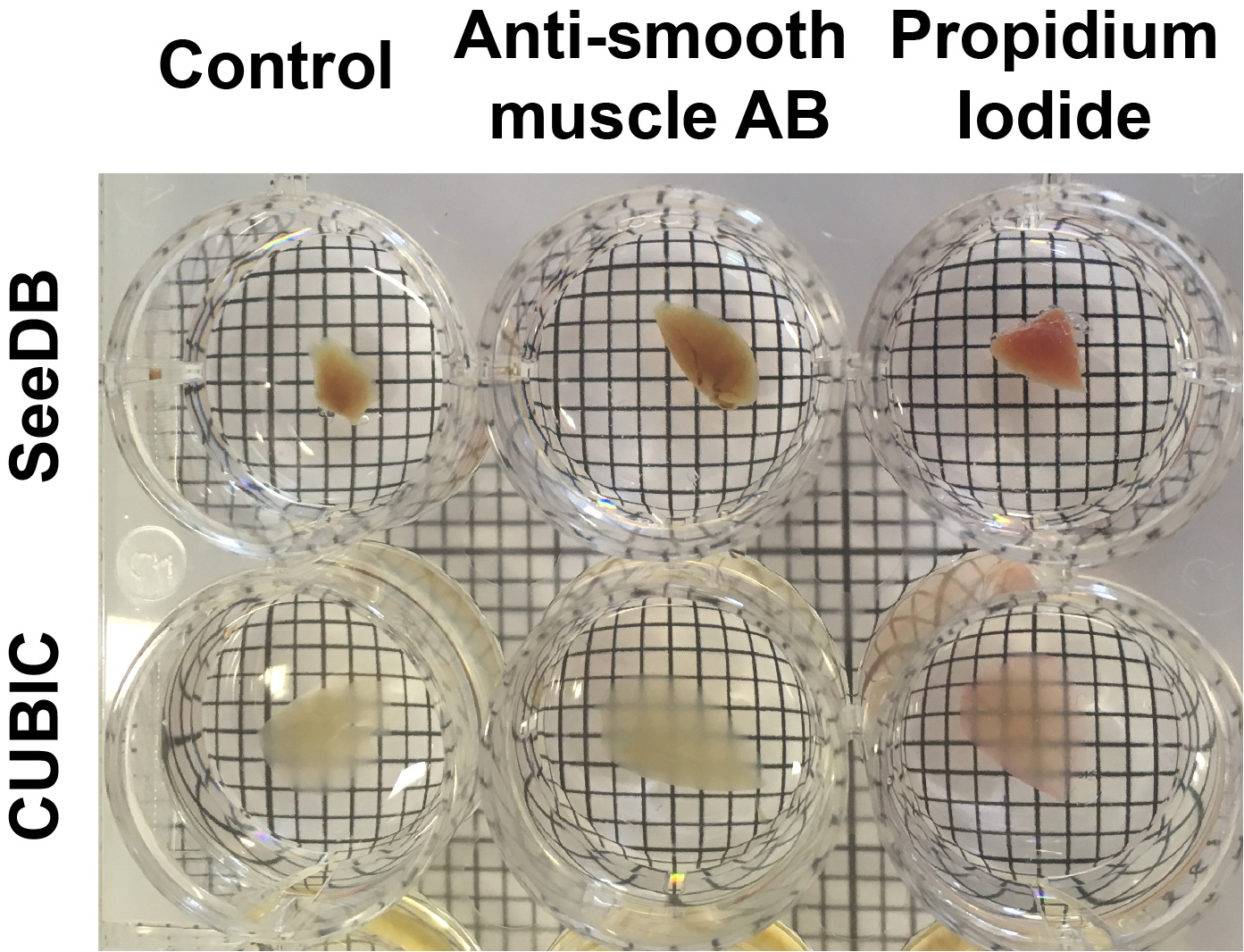


**Supplemental Figure S3:** Transparency of *ex vivo* lung sections after employing different clearing and staining protocols. While the top row is immersed in a fructose solution with a high refractive index (SeeDB), the bottom row is delipidated, decolorized, and immersed in a high refractive index sucrose solution (CUBIC-2). The left column serves as control (unstained), whereas the middle and right columns were stained with anti-alpha smooth muscle actin antibody and propidium iodide, respectively.
